# Meeting Report: The Terabase Metagenomics Workshop and the Vision of an Earth Microbiome Project

**DOI:** 10.4056/sigs.1433550

**Published:** 2010-12-25

**Authors:** Jack A. Gilbert, Folker Meyer, Dion Antonopoulos, Pavan Balaji, C. Titus Brown, Christopher T. Brown, Narayan Desai, Jonathan A Eisen, Dirk Evers, Dawn Field, Wu Feng, Daniel Huson, Janet Jansson, Rob Knight, James Knight, Eugene Kolker, Kostas Konstantindis, Joel Kostka, Nikos Kyrpides, Rachel Mackelprang, Alice McHardy, Christopher Quince, Jeroen Raes, Alexander Sczyrba, Ashley Shade, Rick Stevens

**Affiliations:** 1 Argonne National Laboratory, Argonne, IL USA; 2 Department of Ecology and Evolution, University of Chicago, Chicago, USA; 3 College of Engineering, Michigan State University, East Lansing, MI, USA; 4 Department of Microbiology and Cell Science, University of Florida, Gainesville, FL, USA; 5 Department of Evolution and Ecology, University of California, Davis, USA; 6 DOE Joint Genome Institute, Walnut Creek, CA USA; 7 Illumina, Saffron Walden, U.K.; 8 NERC Centre for Ecology and Hydrology, Oxfordshire,U.K.; 9 Department of Computer Science and Department of Electrical & Computer Engineering, Virginia Tech, Blacksburg, VA USA; 10 Department of Cancer Biology and Translational Science Institute, Wake Forest University Winston-Salem, USA; 11 Center for Bioinformatics ZBIT, Tübingen University,Tübingen, Germany,; 12 Lawrence Berkeley National Laboratory, Earth Sciences Division Berkeley, CA USA; 13 Department of Chemistry and Biochemistry, Boulder, CO, USA; 14 Roche-454, Branford, CT USA; 15 Seattle Children’s Hospital, Seattle, WA USA; 16 School of Civil and Environmental Engineering and School of Biology, Georgia Institute of Technology, Atlanta, GA, USA; 17 The Florida State University, Tallahassee, FL USA; 18 Department of Algorithmic Bioinformatics, Heinrich-Heine University, Düsseldorf, Germany.; 19 Max-Planck-Institut fur Informatik, Max-Planck Research Group for Computational Genomics and Epidemiology, Saarbrücken, Germany; 20 Department of Civil Engineering, University of Glasgow, Glasgow U.K.; 21 Department of Molecular and Cellular Interactions, VIB - University of Brussels, Brussels, Belgium.; 22 Department of Molecular, Cellular and Developmental Biology, Yale University, New Haven, CT USA

## Abstract

Between July 18^th^ and 24^th^ 2010, 26 leading microbial ecology, computation, bioinformatics and statistics researchers came together in Snowbird, Utah (USA) to discuss the challenge of how to best characterize the microbial world using next-generation sequencing technologies. The meeting was entitled “Terabase Metagenomics” and was sponsored by the Institute for Computing in Science (ICiS) summer 2010 workshop program. The aim of the workshop was to explore the fundamental questions relating to microbial ecology that could be addressed using advances in sequencing potential. Technological advances in next-generation sequencing platforms such as the Illumina HiSeq 2000 can generate in excess of 250 billion base pairs of genetic information in 8 days. Thus, the generation of a trillion base pairs of genetic information is becoming a routine matter. The main outcome from this meeting was the birth of a concept and practical approach to exploring microbial life on earth, the Earth Microbiome Project (EMP). Here we briefly describe the highlights of this meeting and provide an overview of the EMP concept and how it can be applied to exploration of the microbiome of each ecosystem on this planet.

## Introduction

The grand challenge of microbial ecology is to understand microbes (bacterial, archaeal, eukaryal and viral) in terms of who they are and what they do. Between July 18^th^ and 24^th^ 2010, 26 leading researchers from microbial ecology, bioinformatics and computing came together in Snowbird, Utah (USA) to discuss this challenge. The “Terabase Metagenomics” and was sponsored by the Institute for Computing In Science (ICiS) summer 2010 workshop program.

The aim of this extended workshop was to explore fundamental questions of microbial ecology that could be addressed using advances in sequencing potential. In particular, participants were challenged with the idea that technological advances in next-generation sequencing platforms such as the Illumina HiSeq 2000 can generate in excess of 250 billion base pairs of genetic information in 8 days. Thus, the generation of a trillion base pairs of genetic information is becoming a routine matter. Participants were requested to brainstorm about the best possible use of this type of information. As a result of three charge presentations by the organizers, and in depth discussions, the main outcome from this meeting was the birth of a concept and practical approach to exploring microbial life on earth. Here we describe the highlights of this meeting and overview the rationale and design of a proposed Earth Microbiome Project (EMP).

## Charge Presentations and Structure of the Meeting

The meeting was arranged around a group discussion and break-out sessions, with the attendees and organizers meeting each day to discuss the issues in a informal style. However, during the six day meeting, several inspirational talks were given that demonstrated the current state-of-the-art in examining microbial ecology and to provide some themes for the future against which to structure discussions. The first talk was given by **Rick Stevens (Argonne National Laboratory),** who provided us with the central tenet of the meeting: to ask ourselves what science we would do, and what microbial ecology questions we could answer if we had access to a trillion base pair sequencing run. In essence, if we had unlimited funds, what would we do? Rick outlined the fundamental questions the guided the meeting such as “Does everything have the potential to be everywhere?” and “How are microbes distributed across the planet?”

On Day 2 **Jack Gilbert** and **Folker Meyer (Argonne National Laboratory)** gave presentations regarding mega-sequencing projects: turning data into information, and computational infrastructure: why compute twice when you only have to do it once. These laid the foundation for addressing which ecological questions we can answer and what research we can do with sequencing data given current technology (including computational infrastructure).

On Day 3 **Rachel Mackelprang** and **Rob Knight (University of Colorado)** gave talks that highlighted the role of genomes in structure metagenomic data, and analyzing 16S rRNA data on a massively parallel scale. Again, examples of projects were given and more questions that remain unresolved were highlighted. **Jonathan Eisen (University of California, Davis)** and **Jeroen Raes (University of Brussels)** gave the final inspirational talks, which discussed problems with annotation and bioinformatics challenges associated with current and future mega-sequencing projects. These talks laid the groundwork for the implementation discussions that ensued. The discussions were wide-ranging, often running late into the night. Importantly we were often found discussing the issues outside of a traditional conference room, taking full advantage of the amazing landscapes that Snowbird had to offer.

On Days 4 and 5, the discussions were focused entirely on laying out the questions that could be answered with terabase pair sequencing, the products which could be developed on an international scale to enhance data analysis and the many varied problems from sampling acquisition to DNA extraction and potential biases.

## The Scope of the Challenge of Characterizing the Microbial World

There are approximately 1 x 10^30^ microbial cells on earth. The average quantity of DNA in each of the cells is ~10 million base pairs. To date, the total global environmental DNA sequencing effort has produced less than 1 percent of the total DNA found in a liter of seawater or a gram of soil. Hence, we have vastly under-sampled the complexity and diversity of microbial life on Earth. Recent advances in high-throughput sequencing technologies have provided an unprecedented opportunity to explore the microbial universe.

## Outcomes: The Earth Microbiome Project

To structure our response to challenges and to help define specific questions and answers, over the last two days the attendees were divided up into specific groups. Each group was given a topic to respond to and tasked with writing up the response. A total of eight topics were assigned, namely: What is the project we are describing? What are the current limitations of microbial community analysis? Why will this project be novel, and why will it succeed? Who are the main beneficiaries of the project and its deliverables? How will the project enable predictive modeling of microbial communities? How much sampling and sequencing will be needed to answer the test specific hypotheses? How much will the project cost? What are the potential risks of the project? To start, the project was given a name; the group decided to pay homage to the Human Microbiome Project and the grandiose nature of the proposed research. In this way, The Earth Microbiome Project was born.

The Earth Microbiome Project (www.earthmicrobiome.org) presents a revolution in how we tackle the challenge of understanding the interactions among microbes and their environments, and defines both questions and a potential suite of tools to provide answers. We wish to sequence microbes and microbial communities from a broad range of biomes (an environment with unique environmental parameters, e.g. a hydrothermal vent on an abyssal plain) to achieve three main goals. First, to define microbial community structure, and to explore the factors that affect community structure at different scales. Second, to explore the protein universe and attempt to produce a complete inventory of protein family diversity. Finally, to curate this information to create a global database of samples, genes and proteins the can be used to answer fundamental questions about the ecology of life on and off the earth.

As envisioned, the Earth Microbiome Project would be a massively multidisciplinary effort to analyze microbial communities across the globe. The general premise is to examine microbial communities from their own perspective, which is formed by their immediate environments. This means that, from the perspective of a microbe the world is a very different place, it is sensed only by availability of nutrients and favorable environmental conditions, therefore, this is the way in which we should structure our attempt to explore their world. Hence we propose to characterize the Earth by environmental parameter space relevant to microbes, and then to explore these different biomes using samples currently available from researchers across the globe. We will analyze these communities using metagenomics, metatranscriptomics and amplicon sequencing to produce a global Gene Atlas describing protein space, environmental metabolic models for each biome, approximately 500,000 microbial genomes, and a data-analysis portal for visualization of all information.

To achieve these general aims, the EMP will focus on ten core questions which can be grouped into different sections:

**Section 1 - Community Structure:**
1. Are microbial communities structured primarily by environmental conditions or trophic/metabolic interactions?
2. If microbes are structured by environmental conditions, how do we define the Environmental Parameter Space (EPS) to characterize microbiomes?
3. What are the primary mechanisms of cross-kingdom interaction, metabolic or genetic?

**Section 2 - Defining Physiology and Metabolic Capability:**
1. Is ecosystem function defined by community taxonomy or by the trophic/metabolic dynamics in that ecosystem, i.e. who is doing what, how fast and by what mechanisms?
2. What is the role of rare microorganisms in an ecosystem, e.g. functional plasticity or specific biochemical function?

**Section 3 - Practical Considerations:**
1. How do we sample microbiomes to best explore global structure, e.g. temporal studies, experimentally controlled perturbations, biogeographic studies, and at what density?
2. How do we best use metagenomic data to re-assemble genomes, and what can we learn from this study to improve the yield of novel microbial genomes from metagenomic studies?

**Section 4 - Models and Visualization:**
1. What aspects/metrics of microbial community structure is it necessary to measure to enable parameterization of predictive ecological models?
2. At what taxonomic level does the pan-genome operate, and what controls this?
3. How do we most accurately visualize global microbial space, and what can this tell us about extraterrestrial microbial communities and fundamental ecology?

To date, virtually all studies have leveraged modest sequencing efforts against small numbers of environments, yet still yielding impressive returns of novel proteins and taxa. The Environmental Parameter Space (EPS) will cover many diverse environments, including marine (water, sediment, and host associated), freshwater (rivers, lakes, etc.), terrestrial (surface, subsurface, rock, etc.), air (particle associated, rain water associated, etc.), extreme environments (extremes of temperature, salinity, pH, UV exposure, desiccation, pressure, etc.) and manmade locations (human interactive environments, cities, agricultural practice areas, transportation, etc.). However, environmental samples will not be the sole aim. We will also explore lab-based mesocosm and microcosm studies in which environmental manipulation will enable us to identify microbial community dynamics (e.g. Winogradsky columns). We propose a systematic re-evaluation and characterization of microbial studies relevant to the global scale analysis of protein space, and microbial diversity with the following tasks:

1. **Defining Environmental Parameter Space (EPS)**. Microbes live in EPS and tend to show similar functional and taxonomic properties when they are isolated from similar environments. We will define microbial communities by their position in EPS.
2. **Defining the Ideal Sampling Strategy**. EPS-defined biomes will be used to describe an “ideal” sampling strategy, i.e. one that provides enough breadth and depth to best determine the full extent of the protein universe, to explore trophic level interactions in microbial communities, and to facilitate generalization of results across biomes and across spatial and temporal scales (to allow for predictive modeling). We recognize that even 200,000 samples will merely scratch the surface of microbial diversity: the goal of this project is not to characterize all microbial life on the planet, but to provide a framework for assisting in interpreting and integrating vast numbers of other studies as sequencing increasingly becomes commoditized.
3. **Defining the Realistic Sampling Strategy**. We intend to catalogue the Global Environmental Sample Inventory (GESI) derived from thousands of researchers. We will only use samples that adhere to the Minimal Informatic about a Metagenomic Sequence (MIMS) [1] and an Environmental Sequence (MIENS) [2] standards, and will determine the extent to which the currently available GESI fulfills the “ideal” sampling strategy. This topic was covered in a closed meeting of the EMP advisory committee in October 2010 [3].
4. **EMP Sequencing Strategy.** The GESI will be sequenced strategically based on priority samples that best fit the ‘ideal’ sampling strategy. This task will involve ~200,000 sampling locations over 3 years; from each we will sequence ~30 million reads (~6 billion base pairs) divided unequally between total community DNA (metagenomics), mRNA (metatranscriptomics) and amplified marker-genes (e.g. 16S rRNA).
5. **EMP Deep Sequencing Strategy of Selected Environments.** We will pick 100 samples against which to leverage 60 billion reads (12 Trillion base pairs) per sample spread across 4 replicates with metagenomics, metatranscriptomics and amplified gene-markers.
6. **Application of a Standard Data Analysis Pipeline.** Analysis of that data including metrics of diversity, richness, statistical similarity, non-parametric analyses are essential to provide a resource for the community. We will compute Alpha- (within-sample) and Beta- (between samples/over time or space) diversity indices, protein annotation and conserved domain prediction, 3-D protein structure prediction, genome assembly from metagenomic data, functional metabolic modeling (modelSEED/Relative Metabolic Flux analysis), non-parametric statistical analysis (canonical correlations, network mapping, ANOSIM, dendrogram clustering from dissimilarity matrices, etc.).
7. **Genome Analysis.** We estimate that from the ~2.4 quadrillion base pairs of sequencing data we could assemble over 500,000 microbial genomes, helping to answer many questions regarding microbial evolution, the pan-genome concept, and horizontal gene transfer.

The Earth Microbiome Project will have many deliverables. We believe that, like previous mega-sequencing projects, the data provided here will produce spin-out research which will make significant contributions to our understanding of microbial ecology. We cannot know or implement the requirements of every researcher; hence, we aim to provide the data, and analysis of the data, in a format which is widely accessible. The following key deliverables will be of considerable benefit to a wide number of communities: **Gene Atlas (GA)** – a centralized repository and database for all sequencing and metadata information acquired during this study. **Earth Microbiome Assembled Genomes (EM-AG)** – all metagenome-derived assembled microbial genomes will be deposited in public repositories. **Earth Microbiome VIsualisation Portal (EM-VIP)** –we want to view the Earth from the perspective of microbes, describing environmental parameter space and genomic functional space. **Earth Microbiome Metabolic Reconstruction (EMMR)** – based on metagenomic metabolome description and prediction (e.g. modelSEED and Relative Metabolic Flux) we will describe changes in metabolite profiles between all samples. We will deliver descriptions regarding metabolite production in specific biomes, providing another metric against which to refine biome descriptions.

The Earth Microbiome Project (EMP) must be, of necessity, a cross-discipline effort, involving microbial ecologists, genomicists, microbiologists, physicists, computer scientists, mathematicians, and ecosystem modelers, to provide the most comprehensive global assessment of microbial life ever seen. Additionally, similar to the Human Genome Project, which has revolutionized biomedicine, the proposed Earth Microbiome Project will revolutionize the way we can assess and model the health of our changing planet. This has implications for global change science, and understanding the intimate connections between the machinery of our planet- the microbes- and their ecosystems.
